# Rapid identification of a stripe rust resistant gene in a space-induced wheat mutant using specific locus amplified fragment (SLAF) sequencing

**DOI:** 10.1038/s41598-018-21489-5

**Published:** 2018-02-15

**Authors:** Jun-liang Yin, Zheng-wu Fang, Cai Sun, Peng Zhang, Xing Zhang, Chen Lu, Shu-ping Wang, Dong-fang Ma, Yong-xing Zhu

**Affiliations:** 1 College of Agriculture/Hubei Key Laboratory of Waterlogging Disaster and Agricultural Use of Wetland, Yangtze University, Jingzhou, Hubei 434025 China; 2 College of Horticulture and Gardening, Yangtze University, Jingzhou, Hubei 434025 China; 3 Institute of Plant Protection, Chinese Academic of Agricultural Sciences, Beijing, 100093 China; 4 Centro Nacional de Biotecnología (CSIC), Campus Universidad Autόnoma de Madrid, Madrid, 28049 Spain; 5 Hubei Collaborative Innovation Center for Grain Industry, Yangtze University, Jingzhou Hubei, 434025 China

## Abstract

Stripe rust, caused by *Puccinia striiformis* f. sp. *tritici* (*Pst*), is one of the most devastating diseases of wheat. Resistant cultivars are the preferred strategy to control the disease. Space-induced wheat mutant R39 has adult-plant resistance (APR) to *Pst*. Genetic analysis indicated that a single recessive gene, designated *YrR39*, was responsible for the APR of R39 to *Pst*. Bulked segregant analysis (BSA) combined with a SLAF sequencing (SLAF-seq) strategy was used to fine-map *YrR39* to a 17.39 Mb segment on chromosome 4B. The region was confirmed by analysis with simple sequence repeat (SSR) markers. A total of 126 genes were annotated in the region and 21 genes with annotations associated with disease response were selected for further qRT-PCR analysis. The candidate gene *Traes_4BS_C868349E1* (annotated as an F-box/LRR-repeat protein) was up-regulated after 12, 24, 48, and 96 hours post inoculation with *Pst*, suggesting it is likely involved in the resistance. The current study demonstrated that BSA combined with SLAF-seq for SNP discovery is an efficient approach for mapping and identifying candidate functional gene.

## Introduction

Common wheat (*Triticum aestivum* L.) is one of the most important crops worldwide, feeding about one-third of the world population^1^. Unfortunately, sustainability of production is seriously threatened by stripe rust caused by *Puccinia striiformis*. f. sp. *tritici*^2^. When weather conditions are favorable for disease development and spread, yield losses in susceptible cultivars can reach 30 to 50%, and even 100% on highly susceptible cultivars growing in highly favorable conditions^3^. China represents the largest epiphytotic region in the world and wheat crop is regularly threatened by the disease, especially the northwestern and southwestern wheat production zones^4^. Historically, destructive *Pst* epiphytotics occurred in 1950, 1964, 1990 and 2002, causing serious yield losses of 6.0, 3.2, 1.8 and 1.3 million tonnes, respectively, indicating the necessity of control^2^.

Breeding and growing resistant cultivars is the most economic, effective and environmentally friendly approach to control *Pst*^5^. Expression of rust resistance is usually described as all-stage resistance (ASR) effective in seedlings as well as adult plants, and adult-plant resistance (APR)^6^. ASR is usually conferred by single highly effective genes, which are prone to be overcome by new (or previously rare) virulent races^7^. In contrast, APR is more likely to be non-race specific and durable. Nevertheless, it is usually quantitatively inherited, and its protective level is usually incomplete and is affected by growth stage, temperature, humidity and inoculum load^3,8,9^. Considering the advantages and disadvantages of both types, a breeding strategy that combines them is recommended for sustainable control^10^. Therefore, it is important to identify and map more *Pst* resistance genes of both types.

Traditional map based cloning of genes of agronomic importance requiring genotyping of large numbers of individuals in segregating populations, which is time-consuming and labor-intensive, is rapidly being replaced by newer molecular methods such as specific locus amplified fragment sequencing (SLAF-seq), a strategy combining bulked segregant analysis (BSA) and next-generation sequencing (NGS)^11^. For example, Liang *et al*.^12^ quickly mapped an aphid resistance QTL to a 0.31 Mb region in cucumber chromosome 5 by using SLAF-seq + BSA. Jia *et al*.^13^ used a SLAF-seq strategy to develop SNP makers to fine-map the barley *ari*-*e* gene from a previously estimated 10 Mb region to a 0.58 Mb interval. Similarly, genes controlling agronomic traits, such as fruit length and flesh thickness in cucumber^14,15^, dwarfness in *Lagerstroemia*^16^, pericarp color in gourd^17^, flowering-time in orchardgrass^18^, and low-tiller in rice^19^ have been genetically mapped using SLAF-seq + BSA. However, until now, SLAF-seq has not been used to map genes for *Pst* resistance gene in wheat.

In September 2005, common wheat cultivar Zhengmai 9023 had went through a space mutation project. Du *et al*.^20^ then performed the system field evaluation of the mutant progenies and found improved adult-plant *Pst* resistance in several lines. Lv *et al*.^21^ further evaluated the advance generation of resistant lines with *Pst* races CYR32, CYR33 and V26/G22 and found these lines to be stable for APR. In this study, an eighth generation of Zhengmai 9023 space mutation progeny, named R39, was resistance evaluated by seven *Pst* races at seedling and adult-plant stage, which confirmed us the adult-resistance of R39 to these races. Genetic analysis further revealed that R39 carries a recessive gene for APR to *Pst* race CYR33, and then used BSA and NGS-based SLAF-seq to locate the gene to a 17.39 Mb region on chromosome 4B.

## Results

### Wheat mutant R39 has APR to stripe rust

**Figure 1:**
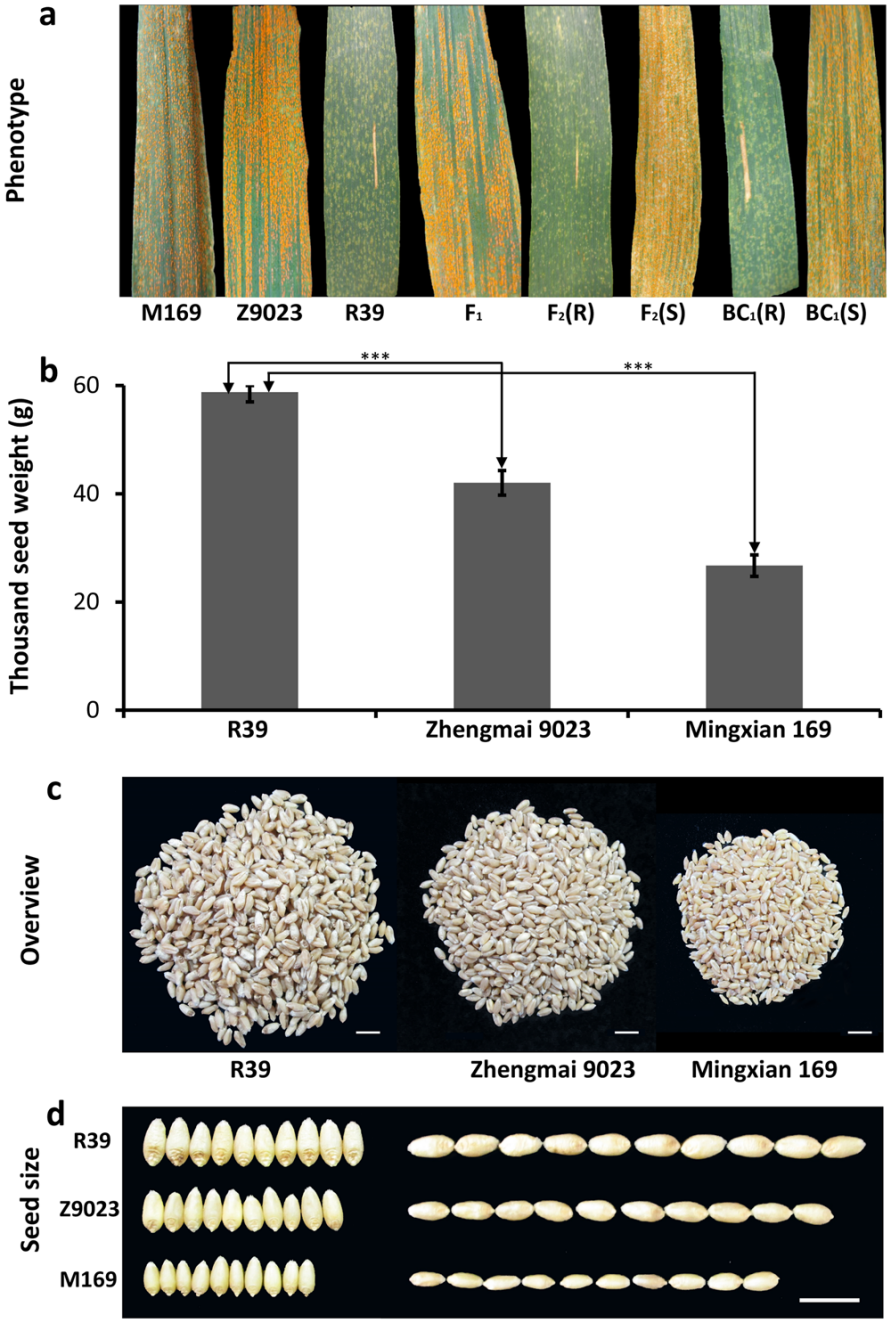
Resistance and production evaluation. R39, stripe rust resistant mutant; M169, susceptible parent Mingxian 169; Z9023, Zhengmai 9023. (**a**) Resistant and susceptible phenotypes of parents and hybrid generations inoculated with *Pst* race CYR33; (R), resistant plant; (S), susceptible plant. (**b**) Thousand seed weight (TSW) of R39, Zhengmai 9023 and Mingxian 169 under field evaluation (*p* < 0.005, n = 3, error bars are SE). (**c**) Overview of 1000-seeds (scale bar, 1 cm). (**d**) Width and length of 10 representative seeds (scale bar, 1 cm).

Seven *Pst* races, Su11-4, Su11-11, CYR29, CYR30, CYR31, CYR32 and CYR33, were used to evaluate the resistance performance of R39 at seedling and adult-plant stage. R39 was susceptible to all seven *Pst* races (IT 3^+^ to 4) at the seedling stage, but was resistant to seven *Pst* races (IT 0; to 1^+^) at the adult-plant stage (Fig. 1a, Supplementary Table S1). However, Zhengmai 9023 and Mingxian 169 were susceptible to seven *Pst* races (IT 3^+^ to 4) at both seedling and adult-plant stage. Besides, TSW of R39 was significant higher than Zhengmai 9023 and Mingxian 169 in field evaluation, suggesting a higher grain production and better seed quality of R39 under *Pst* disease (Fig. 1b–d).

### A recessive gene controls the APR of R39 to CYR33

As shown in Table 1, R39 was resistant (IT 0;), whereas Mingxian 169 was susceptible (IT 4), and F_1_ plants were also susceptible (IT 3–4). The F_2_ population segregated 1 resistant: 3 susceptible and the BC_1_ population segregated 1: 1, respectively, suggesting that resistance was conferred by a single recessive gene (Table 1). The clear differences in response of the parents, F1 individuals, and contrasting resistant and susceptible plants in F_2_ and BC_1_ are shown in Fig. 1. The resistance gene was tentatively designated as *YrR39*. A striking feature of R39 and resistant F_2_ and BC_1_ plants was the leaf spotting (Fig. 1a). This condition was also recessive and completely associated with the resistance phenotype in segregating populations and segregated as a recessive gene in non-inoculated populations (Table 1).

**Table 1:** Frequencies of resistant and susceptible plants in the parents and segregating populations.

Parents and cross^1^	Generation	Resistant/Spotty	Susceptible/No spotty	Expected ratio	*χ*^2^_0.05_(3.84)	*P*
R39 (P_1_)	P_1_	20	0			
Mingxian 169 (P_2_)	P_2_	0	20			
P_1_/P_2_	F_1_	0	15			
P_1_/P_2_	F_2_	120	342	1:3	0.18	0.63
P_1_/P_2_//P_1_	BC_1_	20	19	1:1	0	0.62

^1^P_1_: male parent; P_2_: female parent; plants with IT 0 to 2^+^ were considered resistant and plants with IT 3^−^ to 4 were susceptible.

### Analysis of SLAF-seq data and SLAF tags

Following SLAF library construction and high-throughput sequencing, more than 301 million 100 bp reads were obtained. Most of the bases (90.05%) were of high quality, with quality scores exceeding 30 (Table 2). The SLAF numbers were 420,531 for R39 and 433,264 for Mingxian 169. The average depths of the SLAF markers were 27.02-fold in R39, 34.05-fold in Mingxian 169, 62.07-fold in the resistant pool, and 58.37-fold in the susceptible pool (Table 2). And 40,735 of these tags were polymorphic, with a polymorphism rate of 8.49%.

**Table 2:** Summary of the sequencing data for parental lines and pooled F_2_ plants.

Sample	R39	Mingxian 169	Resistance pool	Susceptible pool
Total reads	41,264,580	53,560,152	108,268,292	98,066,496
GC percentage	46.42%	46.57%	45.42%	45.13%
Q30 percentage	90.22%	89.35%	90.91%	90.74%
SLAF number	420,531	433,264	469,338	464,490
Total depth	11,361,549	14,949,591	29,130,774	27,111,243
Average depth	27.02	34.50	62.07	58.37
Polymorphic SLAF	35,568	37,067	39,675	40,091

### Target regions were located into chromosome 4B by SNP-based association analysis

**Figure 2:**
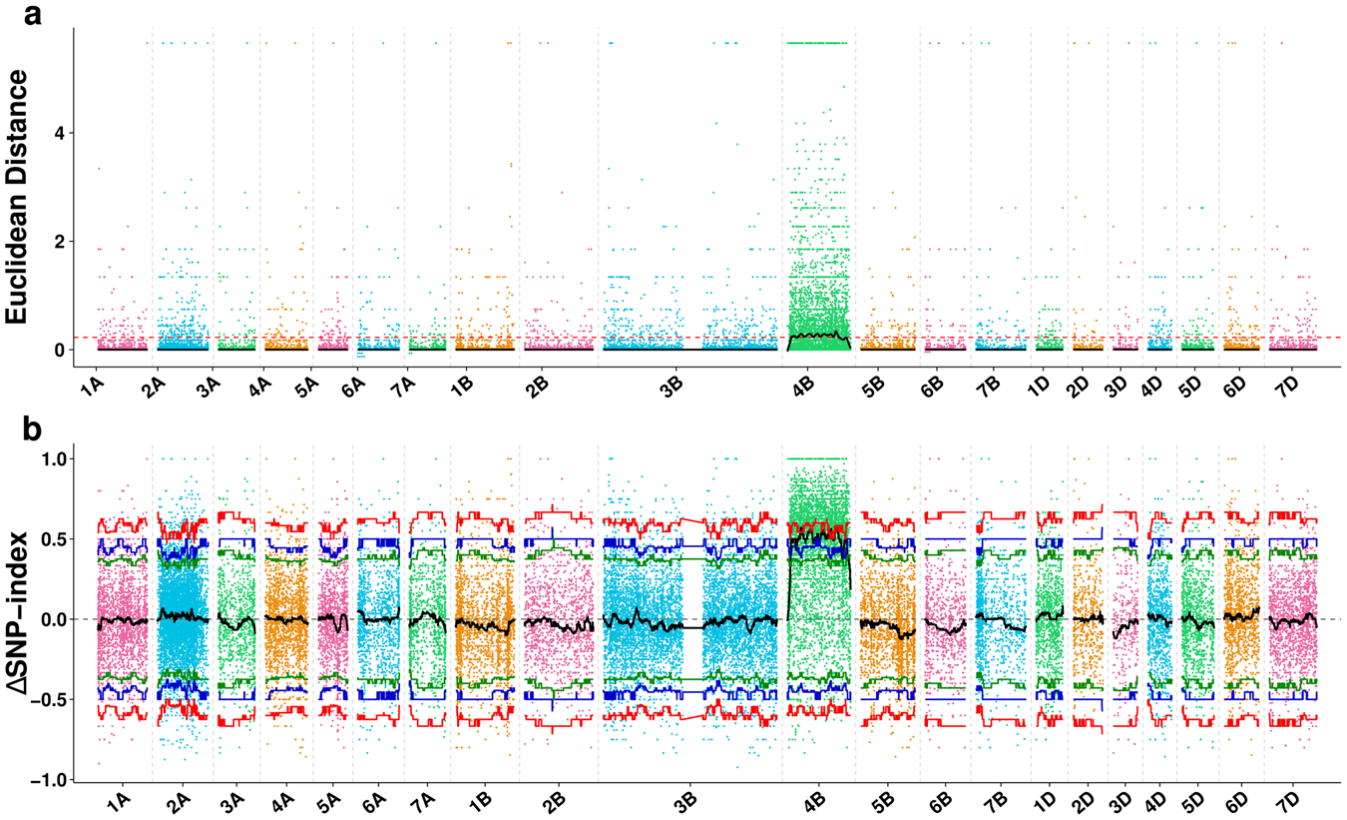
Identification of the target regions through two types of association analysis methods. (**a**) The results of Euclidean Distance association analysis. The x-axis represents the wheat chromosomes and y-axis represents the Euclidean Distance value. The black lines show all fitting results of ED, the red dotted line shows the threshold of ED. The larger the result of ED is, the stronger the association is. The association threshold was 0.23. Peak regions are defined as regions where the Loess-fitted values are greater than the threshold value (*P* < 0.01). (**b**) The results of ΔSNP-index association analysis. The x-axis represents the wheat chromosomes, and the y-axis represents the ΔSNP-index values. Black lines are the average values of ΔSNP-index drawn by sliding window analysis. The red (*P* < 0.01), blue (*P* < 0.05) and green (*P* < 0.1) lines are the threshold values, which was calculated by Loess regression. Peak regions are defined as regions where the smooth value s are greater than the threshold value.

SNP-based association analysis was used to locate the chromosomal region containing *YrR39*. SNPs were firstly called from polymorphic SLAF tags and 394,138 differences were identified between samples and the reference genome. SNPs with (1) multiple alleles, (2) read depths smaller than 4-fold in either bulk, (3) same genotype between R-pool and S-pool, (4) heterozygous genotypes in the recessive parent, and (5) homozygous genotype in the recessive parent but heterozygous genotypes in the recessive bulk, were excluded. Finally, 73,002 high quality SNPs were selected for association analysis. By merging the results from ED (cutoff value: 0.23, *P* < 0.01, Fig. 2a) and SNP-index (*P* < 0.01, Fig. 2b) association analysis, the stripe rust resistance gene *YrR39* was located in a candidate 17.39 Mb region on chromosome 4B (Table 3).

**Table 3:** Information of the association regions.

Chromosome ID	Start	End	Size (Mb)	Gene number
4B	65,532,923	70,614,068	5.08	21
4B	184,932,301	185,577,324	0.65	2
4B	211,728,181	212,587,722	0.86	7
4B	212,968,107	220,850,694	7.88	71
4B	222,261,357	222,345,587	0.08	2
4B	235,321,997	235,499,629	0.18	1
4B	239,484,813	239,920,098	0.44	1
4B	240,575,169	242,606,722	2.03	20
4B	243,058,402	243,251,151	0.19	1
Total	—	—	17.39	126

### Confirmation of the target region by SSR markers

**Figure 3:**
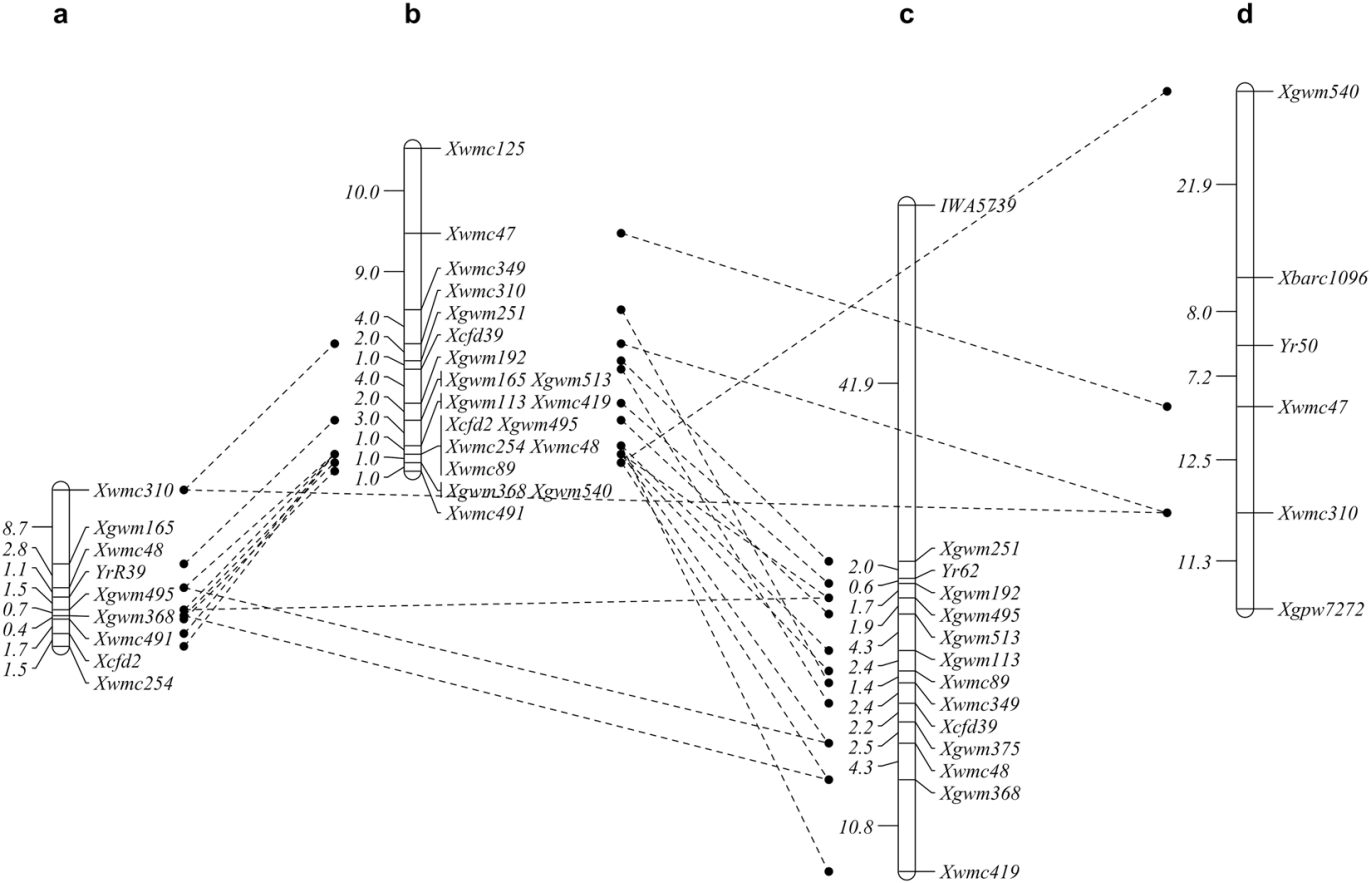
Linkage maps for chromosome 4BL. (**a**) Map position of *YrR39*. (**b)** comparison of the order of SSR markers with those in the consensus map of Somers *et al*.^45^. (**c**) Map position of *Yr62*^9^. (**d**) Map position of *Yr50*^32^.

In total, 83 SSR primers from chromosome 4B were tested for polymorphism and 8 markers showed clear polymorphisms between the resistant and susceptible parents as well as the DNA bulks. Genotype data from 462 F2 individuals were used to construct a genetic map, in which *YrR39* was located a 2.6 cM interval flanked by *Xwmc495* and *Xwmc48* on chromosome 4BL (Fig. 3). This location was consistent with the target regions identified by association analysis and thus supported the region detected by SLAF-seq.

### Influence of stripe rust infection on expression of candidate genes

**Figure 4:**
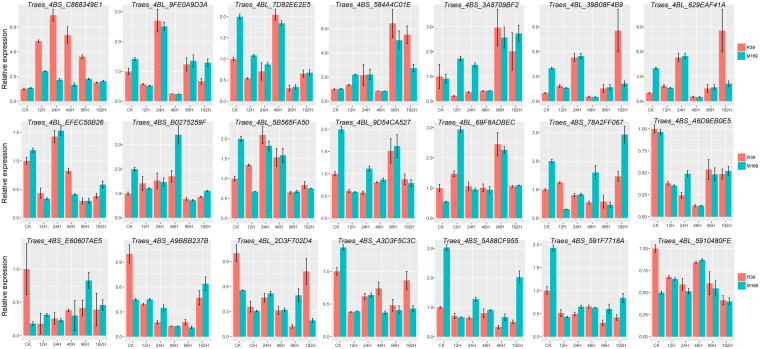
qRT-PCR results of 21 candidate genes detected in the target chromosome region. X-axes represent the time points of 0, 12, 24, 48, 96 and 192 hours post inoculation. Y-axes represent relative expression of the 21 candidate genes in infected leaves of R39 (red bars) and M169 (Mingxian 169, blue bars) at different time points. Each value denotes the mean relative three replicates.

The target regions contained 126 genes (Table 3); 21 of which were annotated as disease resistance or defense response-related genes, WRKY transcription factors, hormone-related genes, LRR or ABC transporters, receptor kinases, protein phosphatases, and/or involved in signal transduction, were selected as potential candidates for confirmation by qRT-PCR assays. The predicted functions of all 21 genes are listed in Supplementary Table S2. According to the qRT-PCR results, candidate gene *Traes_4BS_C868349E1* (annotated as an F-box/LRR-repeat protein) exhibited higher expression levels in R39 than in Mingxian 169 at 12, 24, 48, and 96 hours post inoculation with *Pst*. The other 20 genes exhibited no obvious regularity in expression pattern in response to infection (Fig. 4). These results suggested that *Traes_4BS_C868349E1* was likely involved in the resistance of R39 to stripe rust.

## Discussion

In this study, a classic genetic analysis was performed in an adult-resistance cultivar R39 and results indicated that adult-resistance of R39 to *Pst* race CYR33 was controlled by a recessive gene, which was tentatively designated as *YrR39* (Fig. 1a). Next generation sequencing based SNPs markers development is an effective and high-resolution technique for fine mapping of major genes and QTLs^22^, but this approach has not been frequently used for marker development and isolation of functional genes in common wheat. To rapidly identify *YrR39*, in this study, we used SLAF-seq + BSA approach to analyze two parents and two pooled F2 population samples to detect genomic regions associated with *Pst* resistance in wheat. Finally, *YrR39* was located on chromosome 4B with a size of 17.39 Mb (Fig. 2, Table 3). Considering the fact that only draft genome data of wheat is available at this moment^23^ and wheat is an allohexaploid species with extremely large genomes and high proportion (>80%) of repeated sequences^11,24^, the candidate regions spanning 178 Mb is a fairly acceptable result. Furthermore, classic SSR markers were used to confirm the target regions located by SNP-based association analysis. The constructed linkage map showed that the *YrR39* was delimited to a 2.6 cM region flanked by *Xwmc495* and *Xwmc48* in chromosome 4BL, which further confirmed the accuracy of SLAF-seq (Fig. 3).

Totally, 126 genes were identified from the target regions (Table 3). Based on functional annotations, 21 genes, which were descripted as disease resistance and defense response related genes, WRKY transcription factors, hormone related genes, LRR or ABC transporter, receptor kinase, protein phosphatase, and/or involving in signal transduction^25–30^, were likely to be involved in plant resistance and were selected as good candidates for further analysis (Supplementary Table 2). According to the qRT-PCR results, the expression level of the gene *Traes_4BS_C868349E1*, encoding F-box/LRR protein, was up-regulated in flag leaves of resistant R39 in response to infection (Fig. 4). It is widely accepted that resistance genes with LRR domains have major roles in the regulation of the resistance of plants to pathogens and insects^12^. For example, in tobacco and tomato, van den Burg *et al*.^25^ proposed that ACRE189/ACIF1, a F-Box/LRR protein, which was activated by pathogen recognition to regulate cell death and defense responses, could regulate defense responses via methyl jasmonate- and abscisic acid-responsive genes. In *Arabidopsis*, Yan *et al*.^31^ has proved that F-box/LRR protein COI1 was directly functioning as a jasmonate receptor to involve in defense responses. In the present study, we speculated that *Traes_4BS_C868349E1* may be the key candidate gene responsible for R39 resistance to stripe rust and it may activate wheat defense response by regulating the hormone signaling such as jasmonate and abscisic acid. Elucidation of a more detailed mechanism of *Traes_4BS_C868349E1* in regulating wheat plant defense response will be the subject of future studies.

Until now, *Yr50*, a dominant gene and characterized as seedling resistance to Pst races CYR32 and CYR33, and *Yr62*, a quantitative trait loci characterized as high-temperature adult-plant (HTAP) resistance to *Pst* races PST-116 and PST-117, were mapped to 4BL^9,32^. In contrast, *YrR39* was a recessive gene and characterized as APR to *Pst* race CYR33. Moreover, R39 had a different genetic background from former used cultivars PI 192252 and CH223. Therefore, *YrR39* is different from these two genes and is considered to be a new resistance gene (Fig. 3). Interestingly, a striking feature, the abnormally spotty phenotype, was always observed in the leaves of resistant parent R39, resistant F_2_ and BC_1_ progenies (Fig. 1a), indicating that the spotty trait is also recessive and completely associated with the resistance phenotype in segregating populations and segregated as a recessive gene in non-inoculated populations (Table 1). Although R39 showed a flecking response, its production and seed quality was significantly better than Zhengmai 9023 and Mingxian 169 under field evaluation (Fig. 1b–d). In several pathosystems, lesion mimic mutations have been shown to be involved in programmed cell death, which in some instances leads to enhanced disease resistance to multiple pathogens^33^. Therefore, we speculated that R39 is a lesion mimic mutant and the spotty trait may be responsible for the *Pst* resistance at adult-plant stage. The relationship between spotty trait and resistance is being analyzed at present.

In conclusion, we found that the space-induced mutant R39 showed APR to *Pst* and genetic analysis indicated that a recessive gene was responsible for the adult-resistance to *Pst* race CYR33. Combined of BSA and SLAF-seq method was used and candidate region was located into chromosome 4B with a size of 17.39 Mb. The regions were further confirmed by linkage SSR markers. qRT-PCR results show the gene *Traes_4BS_C868349E1*, encoding F-box/LRR protein, was obviously up-regulated in stripe rust infected R39, but not in Mingxian 169 (susceptible parent), suggesting it is involved in resistance response. Regulating the hormone signaling processes is the possible mechanism of *Traes_4BS_C868349E1* acting wheat defense response.

## Material and Methods

### Plant materials and phenotypic collection

R39 was crossed as male parent with susceptible wheat cultivar Mingxian 169. F_1_, F_2_ and BC_1_ (R39/Mingxian 169//R39) progenies were generated for the study. Plants of the parents, progenies, and Zhengmai 9023 were used in seedling and adult-plant tests in a temperature-controlled greenhouse as described by Zhou *et al*.^2^. Briefly, Seedlings were grown in the greenhouse under controlled conditions. When the first leaves were fully expanded, all seedlings were inoculated with fresh urediniospores of seven *Pst* races (CYR29, CYR30, CYR31, CYR32, CYR33, Su11-4 and Su11-11). After 24 h at 10 °C in dew chambers plants were transferred to an environmentally controlled greenhouse with a daily cycle of 16 h light at 18 °C and 8 h darkness at 10 °C. For adult-plant tests, germinated seedlings were vernalized for 5 weeks in a 4 °C refrigerator, prior to transplanting to pots and grown in a greenhouse. One month later, the parents and progenies plants at the booting to heading stage were inoculated with urediniospores mixed with talc and incubated as described above. Infection type (IT) data were recorded 18–20 days after inoculation based on a 0–4 scale as descripted by Zhou *et al*.^2^. Plants with ITs 0 to 2+ were considered to be resistant and those with ITs 3− to 4 were considered to be susceptible. R39, Zhengmai 9023 and Mingxian 169 were planted in 2016 for field evaluation (Jingzhou, GPS: 112.15028E, 30.362437 N). After harvesting and drying, the seeds were collected to measure thousand seed weight (TSW). Three replicates of each cultivar were performed in this experiment.

### DNA isolation and SLAF library construction for high-throughput sequencing

Young healthy leaves from R39, Mingxian 169 and F_2_ individuals were collected, frozen in liquid nitrogen, and used for DNA extraction. Total genomic DNA was prepared from each plant using the CTAB method^5^. The DNA was quantified using a NanoDrop 2000 spectrophotometer (Thermo Scientific, USA). In total, 45 resistant plants and 45 susceptible plants were selected from the F_2_ population and an equal amount of DNA from each plant in each response group were pooled as the resistant pool (R-pool) and susceptible pool (S-pool) and adjusted to final concentrations of 40 ng/ul. DNA of the parents and pools were digested to completion with *RsaI* (NEB, Nanjing, China). A single-nucleotide A overhang was added to the digested fragments with Klenow Fragment (3′-5′ exo-) (NEB, Nanjing, China) and dATP at 37 °C, and then the Duplex Tag-labeled Sequencing adapters (PAGE purified, Life Technologies, Gaithersburg, MD, USA) were ligated to the A-tailed DNA with T4 DNA ligase. The sequence depth of two parental lines was about 10×, and each pool was about 50×. PCR was performed using diluted shearing-ligation DNA samples, dNTP, Q5® High-Fidelity DNA Polymerase and PCR primers. PCR products were then purified using Agencourt AMPure XP beads (Beckman Coulter, High Wycombe, UK). Fragments ranging from 300 to 500 base pairs (with barcodes and adaptors) in size were excised and purified using a QIAquick gel extraction kit (Qiagen, Hilden, Germany). Gel-purified products were then diluted for pair-end sequencing (each end 100 bp) on an Illumina HiSeq 2500 platform using the standard protocol (Illumina Inc., San Diego, CA, USA) at Beijing Biomarker Technologies Corporation (http://www.biomarker.com.cn).

### Analysis of SLAF-seq data

Low-quality reads (quality score < 30) were filtered out and raw reads were sorted to each progeny according to barcode sequences. After the barcodes were trimmed from each high-quality read, clean reads from the same sample were mapped onto the *Triticum aestivum* L. genome sequence^23^ using Burrows-Wheeler Aligner software^34^. Samtools^35^ was used to mark duplicates, and then GATK^36^ was used for local realignment and base recalibration. A SNP set was formed by combining GATK and Samtools SNP calling analysis with default parameters. SNPs identified between the pools were regarded as polymorphic for association studies. In this study, P and M refer to the male (resistant) and female (susceptible) parents, while ab and aa refer to the R-pool and S-pool, respectively. Two association analysis methods, SNP-index and Euclidean distance (ED), were used.

SNP-index is an association analysis method to find significant differences in genotypic frequency between the pools, indicated by Δ(SNP-index)^37^, which was calculated as:

1 $$\begin{array}{rcl}\mathrm{SNP} \mbox{-} \mathrm{index}({\rm{ab}}) & = & \mathrm{Mab}/({\rm{Pab}}+{\rm{Mab}}),\\ \mathrm{SNP} \mbox{-} \mathrm{index}({\rm{aa}}) & = & \mathrm{Maa}/({\rm{Paa}}+{\rm{Maa}}),\\ {\rm{\Delta }}(\mathrm{SNP} \mbox{-} \mathrm{index}) & = & \mathrm{SNP} \mbox{-} \mathrm{index}({\rm{ab}})-\mathrm{SNP} \mbox{-} \mathrm{index}({\rm{aa}}),\end{array}$$

In which Maa was the depth of the aa population derived from Maa, and Paa was the depth of the aa population derived from P; Mab indicates the depth of ab population derived from Mab, and Pab indicates the depth of ab population derived from P^38^.

Euclidean distance (ED) association analysis is a typical method that calculates Euclidean distance (quadratic sum root of differences between bulks from the depth of four types of base) and is satisfied by ED. Theoretically, the higher the ED value, the closer the object site^38^. ED was calculated as follows: Aaa, Caa, Taa, and Gaa respectively represent the depth of bases A, C, T and G at a site in the susceptible pool, and Aab, Cab, Tab, and Gab represent the depth of bases A, C, T and G at a site in the resistance pool, respectively. To decrease the background noise, the original ED values were then raised to a four power set. Peak regions were defined as regions where the Loess-fitted values are greater than three standard deviations above the genome-wide median^39^.

2 $${\boldsymbol{ED}}=\sqrt{{({\boldsymbol{Aaa}}-{\boldsymbol{Aab}})}^{2}+{({\boldsymbol{Taa}}-{\boldsymbol{Tab}})}^{2}+{({\boldsymbol{Gaa}}-{\boldsymbol{Gab}})}^{2}+{({\boldsymbol{Caa}}-{\boldsymbol{Cab}})}^{2}}$$

### Validation by SSR markers

Genomic DNA from R39, Mingxian 169, and resistant and susceptible bulks were used as PCR templates. Resistant and susceptible bulks used here were constructed by mixing equal amounts of DNA from 10 highly resistant and 10 highly susceptible F_2_ plants (selected from 45 resistant and 45 susceptible F2 plants formerly used in SLAF-seq). SSR marker PCR was conducted as follow: 15 μL reaction mixtures containing 1.5 μL template DNA (25 ng/μL), 1.5 μL of each primer pair (5 mmol/μL), 7.5 μL 2 × SuperTaq PCR Mix with loading dye (Genestar, Beijing, China) and 4.5 μL ddH_2_O. Amplification programs were set as 5 min of denaturation at 94 °C, 35 cycles consisting of 30 s at 94 °C, 45 s at 55 °C, 45 s at 72 °C, followed by a 10 min extension at 72 °C. PCR products were separated in 8% polyacrylamide gels and visualized with silver staining. The banding patterns of the resistant parent, susceptible parent and heterozygotes were denoted as A, B and H, respectively^3^. Linkage analyses and map construction were performed through the computer program Mapmaker, Version 3.0^40^. The linkage map was drawn using MapChart version 2.3^41^.

### Gene annotation

Databases for KOG/COG (Clusters of Orthologous Groups of Proteins), KEGG (Kyoto Encyclopedia of Genes and Genomes), GO (Gene Ontology), Swiss-Prot (A manually annotated and reviewed protein sequence database), Pfam (Protein family), and Nr (NCBI non-redundant protein sequences) were used to perform gene function annotations by BLAST^42^.

### qRT-PCR

Total RNA was isolated from leaves using RNAiso Plus (Takara, Dalian, China). Dried RNA samples were dissolved in DEPC-water to 1 μg/μL using a BioPhotometer Plus spectrophotometer (Eppendorf, Hamburg, Germany). RNA was reverse-transcribed using a Takara PrimeScript^®^ RT reagent kit with a gDNA eraser according to the manufacturer’s specifcations. qRT-PCR was performed using a RealMasterMix (SYBR Green) kit (Tiangen, Beijing, China) according to the manufacturer’s specifications. SYBR Green PCR cycling was performed using an iQ™ 5 multicolour real-time PCR detection system (Bio-Rad, Hercules, CA) with 20 μL samples. PCR primers were designed using Primer Premier 5.0 to avoid conserved regions. Primer sequences are listed in Supplementary Table 1. Wheat *TaEF*-*α* (No. Q03033) was used as internal reference gene. The relative quantities were calculated using the 2^−ΔΔCt^ method^43,44^. Each treatment included three replications, and each replication included two technical replications.

### Data availability statement

All data generated or analysed during this study are included in this published article (and its Supplementary Information files). The datasets generated during and/or analysed during the current study are available from the corresponding author on reasonable request.

## Electronic supplementary material

Supplementary Table S1, S2, S3
