# Supplementary material for: Rapid identification of a stripe rust resistant gene in a space-induced wheat mutant using specific locus amplified fragment (SLAF) sequencing
Source: Sci Rep. 2018 Feb 15;8:3086. doi: 10.1038/s41598-018-21489-5 (PMC5814476; doi:10.1038/s41598-018-21489-5)
Supplement: Supplementary file 1 — Supplementary Table S1, S2, S3 [file 41598_2018_21489_MOESM1_ESM.pdf]

Rapid identification of a stripe rust resistant gene in a space-induced wheat mutant using specific locus amplified fragment (SLAF) sequencing

Jun-liang Yin[1,2], Zheng-wu Fang[1], Cai Sun[1,3], Peng Zhang[1], Xing Zhang[1], Chen Lu[1], Shu-ping Wang[1], Dong-fang Ma[1,3,4,5*], Yong-xing Zhu[2*]

[1]College of Agriculture, Yangtze University, Jingzhou, Hubei 434025, China

[2]College of Horticulture and Gardening, Yangtze University, Jingzhou, Hubei 434025, China

[3]Institute of Plant Protection, Chinese Academic of Agricultural Sciences, Beijing 100093, China

[4]Centro Nacional de Biotecnología (CSIC), Campus Universidad Aut ó noma de Madrid, Madrid 28049, Spain

[5]Hubei Collaborative Innovation Center for Grain Industry, Yangtze University, Jingzhou Hubei 434025, China

***Correspondence:**

Dong-fang Ma: madongfang1984@163.com

Yong-xing Zhu: xbnlzyx@163.com

Supplementary Table S1 Infection types produced by R39, Zhengmai 9023 and Mingxian 169 to seven *Pst* races.

Race	Seedling			Adult		
	R39	Zhengmai 9023	Mingxian 169	R39	Zhengmai 9023	Mingxian 169
Su11-4	4[1]	3	4	1	3	4
Su11-11	4	3	4	1^{+}	3^{+}	4
CYR29	3^{+}	3^{+}	4	$0;^{+}$	3^{+}	4
CYR30	3^{+}	3^{+}	4	0;	3^{+}	4
CYR31	4	4	4	1	3^{+}	4
CYR32	3^{+}	4	4	0;	3^{+}	4
CYR33	3^{+}	4	4	0;	4	4

[1] Plants with IT 0 to 2^{+} were considered to be resistant and plants with IT 3^{-} to 4 were susceptible

Supplementary Table S2 Annotation information of genes.

#Gene_ID	COG_class_annotation	GO_annotation	KEGG_annotation	KOG_class_annotation	Pfam_annotation	Swissprot_annotation	nr_annotation
Traes_4BL_2D3F702D4	Signal transduction mechanisms	Molecular Function: phosphoprotein phosphatase activity (GO:0004721);; Cellular Component: nucleus (GO:0005634);; Biological Process: metabolic process (GO:0008152);; Molecular Function: metal ion binding (GO:0046872);;	--	Signal transduction mechanisms	Protein phosphatase 2C	Probable protein phosphatase 2C 75 OS=Oryza sativa subsp. japonica (Rice) PE=2 SV=1	PREDICTED: probable protein phosphatase 2C 75 [Brachypodium distachyon]
Traes_4BL_629EAF41A	--	Molecular Function: sequence-specific DNA binding transcription factor activity (GO:0003700);; Molecular Function: RNA binding (GO:0003723);; Molecular Function: RNA-directed DNA polymerase activity (GO:0003964);; Molecular Function: aspartic-type endopeptidase activity (GO:0004190);; Molecular Function: protein serine/threonine kinase activity (GO:0004674);; Molecular Function: ATP binding (GO:0005524);; Cellular Component: nucleus (GO:0005634);; Biological Process: RNA-dependent DNA replication (GO:0006278);; Biological Process: DNA recombination (GO:0006310);; Biological Process: regulation of transcription, DNA-templated (GO:0006355);; Biological Process: protein phosphorylation (GO:0006468);; Biological Process: proteolysis (GO:0006508);; Biological Process: defense response (GO:0006952);; Biological Process: DNA integration (GO:0015074);; Cellular Component: integral component of membrane (GO:0016021);; Molecular Function:	--	General function prediction only	--	--	Os07g0510800 [Oryza sativa Japonica Group]

		ADP binding (GO:0043531);; Molecular Function: sequence-specific DNA binding (GO:0043565);;					
Traes_4BL_9FE0A9D3A	--	Molecular Function: protein serine/threonine kinase activity (GO:0004674);; Molecular Function: protein tyrosine kinase activity (GO:0004713);; Molecular Function: ATP binding (GO:0005524);; Cellular Component: plasma membrane (GO:0005886);; Biological Process: protein phosphorylation (GO:0006468);; Biological Process: transmembrane receptor protein tyrosine kinase signaling pathway (GO:0007169);; Cellular Component: integral component of membrane (GO:0016021);;	--	--	--	Probable LRR receptor-like serine/threonine-protein kinase At4g20940 GN=At4g20940 OS=Arabidopsis thaliana (Mouse-ear cress) PE=1 SV=1	Putative inactive receptor kinase [Aegilops tauschii]
Traes_4BL_7D82EE2E5	Posttranslational modification, protein turnover, chaperones	Molecular Function: ATP binding (GO:0005524);; Biological Process: ATP catabolic process (GO:0006200);; Biological Process: rRNA processing (GO:0006364);; Biological Process: tryptophan catabolic process (GO:0006569);; Biological Process: transport (GO:0006810);; Biological Process: aromatic amino acid family biosynthetic process (GO:0009073);; Cellular Component: chloroplast stroma (GO:0009570);; Biological Process: indoleacetic acid biosynthetic process (GO:0009684);; Biological Process: embryo development ending in seed dormancy (GO:0009793);; Biological Process: chloroplast relocation (GO:0009902);; Biological Process: thylakoid membrane organization (GO:0010027);; Biological Process: photosystem II assembly (GO:0010207);; Biological Process: vegetative to reproductive phase transition of meristem (GO:0010228);; Biological Process: iron-sulfur cluster assembly (GO:0016226);; Biological Process: cysteine biosynthetic process (GO:0019344);; Biological Process: glucosinolate biosynthetic process (GO:0019761);; Biological Process: regulation of protein dephosphorylation (GO:0035304);; Molecular Function: ATPase activity, coupled to transmembrane movement of substances (GO:0042626);; Biological Process: transcription from plastid promoter (GO:0042793);; Biological	K09013|2.03974e-177|bdi:100830659|ABC transporter I family member 6, chloroplastic; K09013 Fe-S cluster assembly ATP-binding protein (A)	--	ABC transporter;; AAA domain	ABC transporter I family member 6, chloroplastic (Precursor) OS=Arabidopsis thaliana (Mouse-ear cress) PE=1 SV=1	ABC transporter I family member 6, chloroplastic [Triticum urartu]

		Process: positive regulation of transcription, DNA-templated (GO:0045893);; Biological Process: ovule development (GO:0048481);;					
Traes_4BL_EFEC50B26	--	Molecular Function: sequence-specific DNA binding transcription factor activity (GO:0003700);; Biological Process: regulation of transcription, DNA-templated (GO:0006355);; Cellular Component: plastid (GO:0009536);; Molecular Function: sequence-specific DNA binding (GO:0043565);;	--	--	WRKY DNA -binding domain	Probable WRKY transcription factor 3 GN=WRKY3 OS=Arabidopsis thaliana (Mouse-ear cress) PE=2 SV=1	Putative WRKY transcription factor 4 [Aegilops tauschii]
Traes_4BS_A6D9EB0E5	--	Molecular Function: sequence-specific DNA binding transcription factor activity (GO:0003700);; Biological Process: regulation of transcription, DNA-templated (GO:0006355);; Cellular Component: plastid (GO:0009536);; Molecular Function: sequence-specific DNA binding (GO:0043565);;	--	--	WRKY DNA -binding domain	WRKY transcription factor 55 GN=WRKY55 OS=Arabidopsis thaliana (Mouse-ear cress) PE=2 SV=1	predicted protein [Hordeum vulgare subsp. vulgare]
Traes_4BS_584A4C01E	--	--	--	General function prediction only	zinc-binding in reverse transcriptase	--	Disease resistance protein RGA2 [Aegilops tauschii]
Traes_4BS_3A8709	--	Cellular Component: lytic vacuole (GO:0000323);; Biological Process: response to ethylene (GO:0009723);; Cellular Component: basal plasma membrane (GO:0009925);; Biological Process: positive gravitropism (GO:0009958);; Biological Process: auxin efflux (GO:0010315);; Molecular Function: auxin efflux transmembrane transporter activity (GO:0010329);;	K13947|0|sita:101752791| probable auxin efflux carrier component 1b-like; K13947 auxin efflux carrier family (A)	--	Membrane transport protein	Probable auxin efflux carrier component 1b GN=OsJ_32878 OS=Oryza sativa subsp. japonica (Rice) PE=2 SV=1	predicted protein [Hordeum vulgare subsp. vulgare]

BF2		Cellular Component: integral component of membrane (GO:0016021);; Biological Process: transmembrane transport (GO:0055085);;					
Traes_4BS_A3D3F5C3C	Signal transduction mechanisms	Molecular Function: protein serine/threonine phosphatase activity (GO:0004722);; Biological Process: protein dephosphorylation (GO:0006470);; Cellular Component: plastid (GO:0009536);; Molecular Function: metal ion binding (GO:0046872);;	K17506|3.79431e-135|bdi:100832067|probable protein phosphatase 2C 74; K17506 protein phosphatase 1L [EC:3.1.3.16] (A)	Signal transduction mechanisms	Protein phosphatase 2C;; Protein phosphatase 2C	Probable protein phosphatase 2C 74 OS=Oryza sativa subsp. japonica (Rice) PE=3 SV=1	hypothetical protein F775_43512 [Aegilops tauschii]
Traes_4BS_E60607AE5	Function unknown	Biological Process: defense response (GO:0006952);; Molecular Function: ADP binding (GO:0043531);;	K13457|0|bdi:100832592|disease resistance protein RPM1-like; K13457 disease resistance protein RPM1 (A)	Signal transduction mechanisms	NB-ARC domain;; Leucine Rich repeats (2 copies);; Leucine rich repeat	Disease resistance protein RPM1 GN=F17A9.20 OS=Arabidopsis thaliana (Mouse-ear cress) PE=1 SV=1	Disease resistance protein RPM1 [Aegilops tauschii]
Traes_4BS_5A88CF955	--	--	--	--	Alpha/beta hydrolase family;; Alpha/beta hydrolase family;; alpha/beta hydrolase fold	Salicylic acid-binding protein 2 GN=SABP2 OS=Nicotiana tabacum (Common tobacco) PE=1 SV=1	hypothetical protein F775_11854 [Aegilops tauschii]
Traes_4BS_A9	Defense mechanisms	Biological Process: drug transmembrane transport (GO:0006855);; Molecular Function: drug transmembrane transporter activity (GO:0015238);; Molecular Function: antiporter activity (GO:0015297);; Cellular Component: membrane (GO:0016020);;	K03327|1.47334e-132|osa:4349644|Os11g0126100; K03327 multidrug resistance protein, MATE family (A)	General function predi	MatE	Protein TRANSPARENT TESTA 12 GN=F17J16_80 OS=Arabidopsis	hypothetical protein OsJ_32799 [Oryza sativa

BBB237B				ction only		thaliana (Mouse-ear cress) PE=2 SV=1	Japonica Group]
Traes_4BS_78A2FF067	--	Molecular Function: protein serine/threonine kinase activity (GO:0004674);; Molecular Function: ATP binding (GO:0005524);; Cellular Component: mitochondrion (GO:0005739);; Biological Process: protein phosphorylation (GO:0006468);;	--	Signal transduction mechanisms	Protein kinase domain;; Protein tyrosine kinase;; Regulator of chromosome condensation (RCC1) repeat	Serine/threonine-protein kinase-like protein CCR4 (Precursor) GN=MCA23.19 OS=Arabidopsis thaliana (Mouse-ear cress) PE=1 SV=1	predicted protein [Hordeum vulgare subsp. vulgare]
Traes_4BS_591F7718A	--	--	--	--	AP2 domain	Ethylene-responsive transcription factor 1B GN=K14B15.15 OS=Arabidopsis thaliana (Mouse-ear cress) PE=1 SV=2	Ethylene-responsive transcription factor 15 [Aegilops tauschii]
Traes_4BS_B0275259F	--	Molecular Function: protein serine/threonine kinase activity (GO:0004674);; Molecular Function: ATP binding (GO:0005524);; Cellular Component: nucleus (GO:0005634);; Cellular Component: mitochondrion (GO:0005739);; Biological Process: protein phosphorylation (GO:0006468);; Biological Process: signal transduction (GO:0007165);; Biological Process: response to abiotic stimulus (GO:0009628);; Biological Process: response to cytokinin (GO:0009735);; Biological Process: response to abscisic acid (GO:0009737);;	--	Signal transduction mechanisms	Protein kinase domain;; Protein tyrosine kinase;; NAF domain;; Kinase-like	CBL-interacting protein kinase 32 GN=CIPK32 OS=Oryza sativa subsp. japonica (Rice) PE=2 SV=2	CBL-interacting protein kinase 32 [Triticum urartu]
Traes_4BS_C86	--	Molecular Function: ubiquitin-protein transferase activity (GO:0004842);; Biological Process: protein glycosylation (GO:0006486);; Biological Process: ubiquitin-dependent protein catabolic process (GO:0006511);; Biological Process: protein ubiquitination (GO:0016567);;	K10268|3.39129e-87|bdi:100843200|F-box/LRR-repeat protein 4; K10268 F-box and leucine-rich repeat protein 2/20 (A)	General function predi	Leucine Rich repeat	F-box/LRR-repeat protein 4 GN=FBL4 OS=Arabidopsis thaliana (Mouse-ear cress) PE=2 SV=1	predicted protein [Hordeum vulgare subsp. vulgare]

8349E1				ction only			
Traes_4BL_39B08F4B9	--	Cellular Component: plastid (GO:0009536);; Biological Process: regulation of ethylene biosynthetic process (GO:0010364);; Biological Process: stem cell division (GO:0017145);; Molecular Function: protein binding, bridging (GO:0030674);; Biological Process: proteasome-mediated ubiquitin-dependent protein catabolic process (GO:0043161);; Biological Process: regulation of post-embryonic root development (GO:2000069);;	--	--	TPR repeat;; Tetratricopeptide repeat;; Tetratricopeptide repeat;; Tetratricopeptide repeat;; Tetratricopeptide repeat;; Tetratricopeptide repeat	Ethylene-overproduction protein 1 GN=ETO1 OS=Arabidopsis thaliana (Mouse-ear cress) PE=1 SV=2	predicted protein [Hordeum vulgare subsp. vulgare]
Traes_4BL_69F8ADBEC	--	Molecular Function: protein kinase activity (GO:0004672);; Molecular Function: ATP binding (GO:0005524);; Biological Process: protein phosphorylation (GO:0006468);; Cellular Component: integral component of membrane (GO:0016021);;	--	--	Protein tyrosine kinase;; Protein kinase domain	Probable LRR receptor-like serine/threonine-protein kinase MRH1 (Precursor) GN=MRH1 OS=Arabidopsis thaliana (Mouse-ear cress) PE=2 SV=1	putative LRR receptor-like serine/threonine-protein kinase MRH1 [Triticum urartu]
Traes_4BL_5B565FA50	--	Biological Process: response to molecule of fungal origin (GO:0002238);; Cellular Component: vacuole (GO:0005773);; Biological Process: response to stress (GO:0006950);;	--	--	Universal stress protein family	--	universal stress protein 1561 [Hordeum vulgare subsp. vulgare]
Traes_4BL_9	Signal transduction	Molecular Function: protein serine/threonine phosphatase activity (GO:0004722);; Cellular Component: nucleus (GO:0005634);; Biological Process: protein dephosphorylation (GO:0006470);; Cellular Component: plastid (GO:0009536);;	K17506|0|bdi:100830459| probable protein phosphatase 2C 32; K17506 protein	Signal transducti	Protein phosphatase 2C;; Protein phosphatase 2C;;	Probable protein phosphatase 2C 32 OS=Oryza sativa subsp.	PREDICTED: probable protein phosphatase

D54CA527	mechanisms	Biological Process: abscisic acid-activated signaling pathway (GO:0009738);; Cellular Component: integral component of membrane (GO:0016021);; Molecular Function: metal ion binding (GO:0046872);;	phosphatase 1L [EC:3.1.3.16] (A)	on mechanisms	Stage II sporulation protein E (SpoIIE)	japonica (Rice) PE=2 SV=1	2C 32 [Brachypodium distachyon]
Traes_4BL_5910480FE	--	Molecular Function: protein serine/threonine kinase activity (GO:0004674);; Molecular Function: protein tyrosine kinase activity (GO:0004713);; Molecular Function: ATP binding (GO:0005524);; Biological Process: protein phosphorylation (GO:0006468);;	K07198|0|bdi:100832804| carbon catabolite-derepressing protein kinase-like; K07198 5'-AMP-activated protein kinase, catalytic alpha subunit [EC:2.7.11.11] (A)	Signal transduction mechanisms	Protein kinase domain;; Protein tyrosine kinase;; Kinase associated domain 1;; Kinase-like;; UBA/TS-N domain	Carbon catabolite-derepressing protein kinase GN=RKIN1 OS=Secale cereale (Rye) PE=2 SV=1	Carbon catabolite-derepressing protein kinase [Aegilops tauschii]

Supplementary Table S3 Primers for qRT-PCR.

Gene ID	Primer code	Forward	Reverse	Product (bp)	Tm (℃)
No. Q03033	TaEF-1α	TGGTGTCATCAAGCCTGGTATGGT	ACTCATGGTGCATCTCAACGGACT	86	60
Traes_4BL_2D3F702D4	Ta1	CATCAGGTGGTTGTCAACATCG	CCAAGGCATCCAAGAGCAAG	124	60
Traes_4BL_629EAF41A	Ta2	GCATTTCCATTCTCATGGTCGT	AGCAGATTTTATAGTGCATCCCG	171	60
Traes_4BL_9FE0A9D3A	Ta3	CTGTCCGTTTCCTTTCTTCTTGT	GTTGCATTGATTCGTTGTTCTTT	140	60
Traes_4BL_7D82EE2E5	Ta4	TAGCGGCTCGTTTGGTGTAGT	CCTTTGTGAGGGTGCTCTTGC	138	60
Traes_4BL_EFEC50B26	Ta5	GGAGACAGCAACTCTGCCACT	CAGGTTTATCCACGGTGAGGG	138	60
Traes_4BS_A6D9EB0E5	Ta6	GCATGGCTTCTGTGGTGTAGTGT	ATAAGGCAAGTTGGCAGAGGAAA	108	60
Traes_4BS_584A4C01E	Ta7	ATCTGCCGTCCAGCATCTATTT	TGCCTGGGAAAGTAATGGTATG	166	60
Traes_4BS_3A8709BF2	Ta8	TACGCTGTGCTGAGAATGTTGG	TACATCCCAGTTGAGGGTCTTG	165	60
Traes_4BS_A3D3F5C3C	Ta9	AACCTTCTCCGTGACACCCTTCT	TTTGGAACTGAATCCTGGTGAGC	98	60
Traes_4BS_E60607AE5	Ta10	ACTGGATGATGTCTGGGAGCAA	CAAGATGACGGTTGGGAGAAGA	134	60
Traes_4BS_5A88CF955	Ta11	CACCGTCCTTTGTAGGTTCATTAG	TTGTTTCAGCAGCAAGAAGGTCAT	106	60
Traes_4BS_A9BBB237B	Ta12	TCCACCTGGTCCCTCTATCCC	CTGCTCCGCCTATCAATGTCC	167	60
Traes_4BS_78A2FF067	Ta13	ATGTCAAACCGACCTGCTGC	GCATTGTGGGTTGCTTCCTC	110	60
Traes_4BS_591F7718A	Ta14	CAGGCTCATGCTGACATGGTG	CTGCTGCTGGTGGCTTCTTCC	71	60
Traes_4BS_B0275259F	Ta15	CTCCGCACGCTTGTAACCTTTCT	AATCCCACATCTTGCTCCATAAC	117	60
Traes_4BS_C868349E1	Ta16	TAAAGCCCAGAGCAGAGGAATG	AGCTGGTGTACTGCAAGCGAGT	157	60
Traes_4BL_39B08F4B9	Ta17	GGTCACGGACTACGATCCTGC	CACGCTCCATCTTGTCCTCCT	170	60
Traes_4BL_69F8ADBEC	Ta18	CCGTCAGTATCTCCAGCAGCAC	CAAGGGATCATCACCTCGTTCA	86	60
Traes_4BL_5B565FA50	Ta19	TTCGACTTGACGATGAGAACGG	CAGTGAGACCAGCGGGATTG	192	60
Traes_4BL_9D54CA527	Ta20	GGCAGAGGTGGTGAGGAAGGT	CAGTGTTGGACACGGTGAGGC	170	60
Traes_4BL_5910480FE	Ta21	GAACCGTCGTCAAATGAGAACTA	GACCAACACTGGAAGCCAATAAC	123	60
